# Hippocampal Deficits in Amyloid-β-Related Rodent Models of Alzheimer’s Disease

**DOI:** 10.3389/fnins.2020.00266

**Published:** 2020-04-07

**Authors:** Yukti Vyas, Johanna M. Montgomery, Juliette E. Cheyne

**Affiliations:** Department of Physiology, Centre for Brain Research, University of Auckland, Auckland, New Zealand

**Keywords:** hippocampus, Alzheheimer’s disease, mouse models, synaptic plasticity, circuit changes

## Abstract

Alzheimer’s disease (AD) is a progressive neurodegenerative disease that is the most common cause of dementia. Symptoms of AD include memory loss, disorientation, mood and behavior changes, confusion, unfounded suspicions, and eventually, difficulty speaking, swallowing, and walking. These symptoms are caused by neuronal degeneration and cell loss that begins in the hippocampus, and later in disease progression spreading to the rest of the brain. While there are some medications that alleviate initial symptoms, there are currently no treatments that stop disease progression. Hippocampal deficits in amyloid-β-related rodent models of AD have revealed synaptic, behavioral and circuit-level defects. These changes in synaptic function, plasticity, neuronal excitability, brain connectivity, and excitation/inhibition imbalance all have profound effects on circuit function, which in turn could exacerbate disease progression. Despite, the wealth of studies on AD pathology we don’t yet have a complete understanding of hippocampal deficits in AD. With the increasing development of *in vivo* recording techniques in awake and freely moving animals, future studies will extend our current knowledge of the mechanisms underpinning how hippocampal function is altered in AD, and aid in progression of treatment strategies that prevent and/or delay AD symptoms.

## Introduction

Alzheimer’s disease (AD) is the most common neurodegenerative disease affecting more than 40 million people worldwide (Alzheimer’s Disease International, 2018). AD is clinically characterized as a progressive impairment of memory and other cognitive functions, eventually leading to dementia and death (Förstl and Kurz, 1999; Holtzman et al., 2011). There are three stages of AD: (1) “preclinical” asymptomatic phase, (2) mild cognitive impairment where the first symptoms including changes in mood and behavior, confusion, and some memory loss become evident, and (3) dementia in which patients demonstrate deficits in multiple cognitive domains that are severe enough to produce loss of function (Förstl and Kurz, 1999; Jack et al., 2010; Sperling et al., 2011). Post-mortem AD brain tissue is characterized by pathological markers including amyloid plaques, tau neurofibrillary tangles, vascular damage from the plaque deposition, and profound neuronal cell loss (Blessed et al., 1968; Katzman and Saitoh, 1991; Selkoe, 1991; for a review see Uylings and de Brabander, 2002). There are currently no cures for AD or dementia, and any treatments available are only palliative, therefore, many groups internationally are working to further understand the pathophysiology of AD in order to develop potential treatment strategies.

The hippocampus is widely studied in AD as this brain region is essential for forming new memories, and the progressive degeneration of neurons in the hippocampus responsible for short-term memory loss is a hallmark effect of AD (West et al., 1994, 2004; Fox et al., 1996). Microscopic changes in the hippocampus also precede behavioral symptomology in AD patients (for a review see Mufson et al., 2015) and mouse models; therefore, this review will focus on hippocampal deficits observed in AD.

There are two categories of AD, the early-onset familial AD generally occurring before 65 years of age, and the late-onset sporadic AD occurring after the age of 65. Data from extensive human genetic, histopathological, biomarker and animal model studies indicates that the 39–42 amino-acid (aa) peptide amyloid-β plays a prime role in the pathogenesis of familial and sporadic AD (Oddo et al., 2003; Haass and Selkoe, 2007; Bertram et al., 2010; Jack et al., 2010, 2013; Bateman et al., 2012). Here we will focus on the pathological effects of amyloid-β in AD. The 42aa amyloid-β protein is a hydrophobic peptide with an ominous tendency to assemble into long-lived polymers, and this excessive accumulation and deposition of amyloid-β is hypothesized to underlie the cascade of events that ultimately lead to cell death (Selkoe, 1991; Hardy and Higgins, 1992; Hardy and Selkoe, 2002). Early-onset AD is associated with mutations in the amyloid precursor protein (APP) gene, the presenilin 1 (PS1), and the presenilin 2 gene, which increases the production of the 42aa isoform of amyloid-β, an isoform more closely associated with the development of amyloid plaques than the shorter isoforms (Goate et al., 1991; Borchelt et al., 1996; Duff et al., 1996; Citron et al., 1997; Steiner et al., 1999). The strongest risk factor of late-onset sporadic AD is the ε4 allele of apolipoprotein E (a protein involved in the fat metabolism), which also significantly increases the burden of amyloid plaques in the brain (Verghese et al., 2011). Although familial AD cases represent only approximately 5% of AD cases, they have been critical for understanding the molecular mechanisms of AD, and importantly, similar mechanisms occur in sporadic AD.

Rodent models of AD have been extensively studied to examine neurological changes and to test therapeutic strategies that cannot directly be tested in humans. A frequently used single mutation AD model is the PDAPP model, expressing the AβPP_V__717__F_ mutation more commonly known as the AβPP_Ind_ mutation (Games et al., 1995). To more accurately replicate the human AD pathology, many mouse AD models have multiple mutations. Double mutant models include the Tg2567 and APP23 mouse models which both express the AβPP_Swe_ (AβPP_K__670__N/M__671__L_) mutation but with different promoters (Hsiao et al., 1996; Sturchler-Pierrat et al., 1997). Other multi-mutation models are the TgCRND8 and J20 mouse models expressing the AβPP_Swe,Ind_ mutations, the APP/PS1 mice with the AβPP_Swe_/PS1_M__146__L_, AβPP_Swe_/PS1_P__264__L_, AβPP_Swe_/PS1_L__166__P_or the APP_S__we_/PS1ΔE9 mutation, the 5xFAD model with AβPP_Swe,Lnd,Flo_/PS1_M__146__L,L__286__V_ mutation, and the 3xTg-AD triple transgenic mouse model with the AβPP_Swe_/Tau_P__301__L_/PS1_M__146__V_ mutations (Holcomb et al., 1998; Mucke et al., 2000; Chishti et al., 2001; Flood et al., 2002; Oddo et al., 2003; Oakley et al., 2006; Radde et al., 2006; Lindström, 2007; Hall and Roberson, 2012; Graybeal et al., 2015). Additional models include transgenic mice expressing the human APP (hAPP), as well as the SAMP8 mouse model that spontaneously develops AD (Mucke et al., 1996; Morley et al., 2000). Although each of these models displays some deficits associated with AD, the difficulty has been generating models where disease progression reaches stage 3 within the shorter lifespan of rodents. For this reason, double and triple knockout models that show faster rates of disease progression are often favored in the field.

While late-stage AD is characterized by profound neuronal loss, more subtle neuronal changes occur early in AD progression including synapse loss and circuit changes, which have been well correlated with cognitive impairments (Davies et al., 1987; Masliah et al., 1991; Terry et al., 1991; for a reviews see Selkoe, 2002; Palop et al., 2006; Palop and Mucke, 2010). Changes in synaptic function, plasticity, neuronal excitability, brain connectivity, and excitation/inhibition imbalance all have profound effects on circuit function, and are thought to exacerbate disease progression in AD. These circuit changes may be crucial targets for slowing or even preventing disease progression before widespread cellular loss has occurred. Changes in circuit function in AD lead to a high co-morbidity with epilepsy, even in the early stages of disease (Vossel et al., 2013; Subota et al., 2017; for reviews see Noebels, 2011; Vossel et al., 2017). Moreover, seizures worsen disease progression (Volicer et al., 1995) whereas anti-epileptic drugs improve memory impairments in individuals with mild cognitive impairment (Bakker et al., 2015).

## Hippocampal Deficits in Amyloid-β-Related Alzheimer’s Disease

### Behavioral Deficits

**FIGURE 1:**
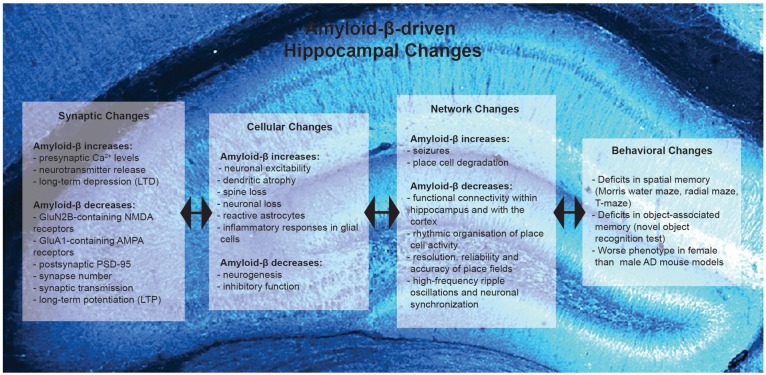
Summary of Amyloid-β-driven hippocampal changes in rodent models of Alzheimer’s disease. Amyloid-β drives changes at the synaptic, cellular, network and behavioral level.

Memory impairments are a major feature of AD that are crucial to replicate in rodents to accurately model the disease. Spatial memory encodes information about ones environment and orientation, and is commonly impaired in AD patients (Henderson et al., 1989; Cherrier et al., 2001). A common behavioral paradigm examining spatial memory in mice is the Morris water maze (MWM). Using the MWM it has been demonstrated that mouse models of AD show impaired spatial memory performance (Chen et al., 2000; Cayzac et al., 2015; Rorabaugh et al., 2017; Bergin et al., 2018). In addition, APP mice require considerably more training to reach the same level of competency as the control mice (Zhao et al., 2014). Furthermore AD-associated behavioral deficits have been observed in other hippocampal-dependent tests such as the radial maze, T-maze, olfactory tubing maze, and in the novel object recognition test (Klevanski et al., 2015; Baranger et al., 2017; Biasibetti et al., 2017). Female 3xTg-AD mice perform worse in working memory tests than males which is correlated with a higher amyloid-β load, and is reflective of the higher prevalence of AD in women (Carroll et al., 2007, 2010). Similarly female APP/PS1/tau triple-transgenic mice perform worse than males in the MWM, which is correlated with increased amyloid-β and tau load (Yang et al., 2018). These studies and others demonstrate that mouse models of AD ubiquitously present hippocampal-dependent behavioral deficits (Figure 1), and also reflect the gender differences in AD prevalence observed in human patients.

### Changes in Glutamatergic Synapse Function

Changes in synapse function are a vital aspect during the early stages of AD progression with amyloid-β playing a complex role as, in addition to its effects on synapses, amyloid-β levels are regulated by synaptic activity. Synapse loss is highly correlated with cognitive impairments in AD (Terry et al., 1991). Synapse loss is also correlated with amyloid-β burden (Terry et al., 1991), and many studies have focused on how amyloid-β influences presynaptic function, postsynaptic receptors and proteins, and consequently synapse function. However, neuronal and synaptic activity also influence the metabolism of amyloid-β (Kamenetz et al., 2003). Furthermore, the extracellular concentration of amyloid-β is critical in determining whether it will aggregate into toxic species (Kamenetz et al., 2003). Consequently, the areas of the brain with the highest basal rates of metabolic and neuronal activity develop the most amyloid-β plaques (Buckner et al., 2005). Synaptic activity rapidly regulates interstitial fluid amyloid-β levels *in vivo* and correlates with local amyloid-β burden (Cirrito et al., 2005). Extracellular amyloid-β levels have been linked to synaptic vesicle release, suggesting that the synaptic amyloid-β levels are regulated presynaptically (Cirrito et al., 2005). Amyloid-β evokes sustained increases in presynaptic Ca^2+^, and acts as a positive endogenous regulator of neurotransmitter vesicle release probability at hippocampal synapses (Abramov et al., 2009). These studies indicate that amyloid-β increases neurotransmitter release and the consequent hyperactivity further leads to more amyloid-β and its subsequent aggregation, resulting in a positive feedback loop (which has been proposed to be a major feature of AD; for a review see Doig, 2018). However, β-amyloid can also lead to depletion of presynaptic proteins involved in neurotransmitter release such as dynamin (Kelly et al., 2005; for a review see Honer, 2003).

Amyloid-β effects multiple postsynaptic proteins and there is evidence that correcting postsynaptic changes can improve impairments in mouse models of AD. More than 90% of synaptic oligomeric amyloid-β is colocalized in the postsynaptic density (Lacor et al., 2004). Amyloid pathology appears to progress in a neurotransmitter-specific manner with the glutamatergic and cholinergic terminals being the most vulnerable, whereas GABAergic terminals appear to be more resilient (for a review see Bell and Claudio Cuello, 2006). In early stages of AD, amyloid-β disrupts neuronal signaling via glutamatergic and acetylcholine receptors (Dougherty et al., 2003; Abramov et al., 2009). Amyloid-β regulates N-methyl-D-aspartate receptor (NMDAR) trafficking (Snyder et al., 2005) and oligomeric amyloid-β leads to a selective loss of GluN2B-containing NMDAR function (Kessels et al., 2013). Increases in the intracellular domain of APP (AICD), which occur in AD, affect NMDAR composition by increasing the prevalence of GluN2B containing receptors (Pousinha et al., 2017). Furthermore, increased ACID reduces excitability of CA1 neurons and impairs spatial memory (Pousinha et al., 2019). Amyloid-β induces NMDAR-dependent degradation of postsynaptic density 95 (PSD-95) at glutamatergic synapses (Roselli et al., 2005). In addition, accumulation of amyloid-β in APP mutant neurons reduces synaptic PSD-95 and GluA1 (Almeida et al., 2005). Interestingly, restoration of PSD-95 levels can rescue memory deficits in AbPP_S__we_/PS1 mice (Bustos et al., 2017). Therefore, amyloid-β also acts postsynaptically to reduce the expression of glutamatergic receptors and proteins, which is directly linked to cognitive impairments in AD.

These amyloid-β-induced pre and post-synaptic alterations consequently impair glutamatergic synaptic transmission in several mouse models of AD. Amyloid-β depresses synaptic transmission, and this was initially noted in APP_Ind_ mice which displayed severe impairments in synaptic transmission between hippocampal CA3 and CA1 cells (Hsia et al., 1999; Kamenetz et al., 2003). Additionally, the APP/PS1 model of AD, which overexpresses mutant human genes for APP and PS1, display deficits in synaptic transmission at a younger age than Tg4510 mice, which overexpress the mutant human Tau gene (Gelman et al., 2018). Therefore, amyloid-β plays a dominant role in causing synaptic deficits in the hippocampus, from the structural to the functional level (Figure 1).

In summary, amyloid-β increases presynaptic transmitter release but its postsynaptic negative effects override these leading to impaired synaptic function in AD. However, many of these studies examined the influence of amyloid-β using via external application *in vitro*. Thus, more *in vivo* studies are required to decipher the influence of intrinsically-released amyloid-β on synapse function, and to understand the temporal relation between AD-associated presynaptic and postsynaptic changes in the hippocampus. Exactly how these complex synaptic changes affect circuit function also remains somewhat a mystery. Nevertheless, disrupted synapse function could directly impact the ability of synapses to undergo synaptic plasticity, which in turn could underlie the memory deficits characteristic of AD.

### Changes in Synaptic Plasticity

Synaptic plasticity has been well studied in AD as deficits in the ability of synapses to undergo changes in strength could be responsible for memory deficits. There is good consensus in the field that impaired synaptic strengthening is a key feature of AD as deficits in long-term potentiation (LTP) occur in many mouse models of AD (Nalbantoglu et al., 1997; Chapman et al., 1999; Gruart et al., 2008; Gengler et al., 2010; Klevanski et al., 2015; Gelman et al., 2018; Viana da Silva et al., 2019). Even transgenic mice expressing only the carboxy-terminal 104 amino acids of APP display deficits in the maintenance of LTP (Nalbantoglu et al., 1997). On the contrary, amyloid-β facilitates synapse weakening in the form of long-term depression (LTD) and depotentiation *in vivo* (Kim et al., 2001). Amyloid-β drives the loss of surface α-amino-3-hydroxy-5-methylisoxazole-4-proprionic acid receptors (AMPAR) by employing signaling pathways of LTD and can also lead to reduced synaptic NMDAR currents (Hsieh et al., 2006; Kessels et al., 2013). The synaptic depression and memory deficits induced by amyloid-β require the AMPAR subunit GluA3, as they are absent in GluA3 knockout mice (Reinders et al., 2016). This suggests that amyloid-β initiates removal of GluA3-containing AMPARs from synapses leading to synaptic and memory deficits (Reinders et al., 2016). Taken together, synaptic strengthening is impaired and synaptic depression is enhanced in AD (Figure 1) (for a review see Mucke and Selkoe, 2012).

Upon investigation of the mechanisms affecting synaptic plasticity in AD, several genes and pathways have been implicated. APP/PS1 mice showed reduced expression of synaptic plasticity genes such as *Arc, Zif268, NR2B, GluR1*, and *Homer-1a* (Dickey et al., 2003). In transgenic mice producing hAPP, dentate granule cells in particular were vulnerable to disruption of Arc expression as well as reductions in actin-binding protein α-actinin-2, which was tightly correlated with reductions in Fos and calbindin, shown previously to reflect learning deficits in these hAPP mice (Palop et al., 2005). Amyloid-β precursor protein (β-APP) fragments and amyloid-β oligomers impair LTP *in vivo*, and in hippocampal slices, this process is mediated via activation of several different kinases, such as c-Jun N-terminal kinase, cyclin-dependent kinase 5, and p38 mitogen-activated protein kinase as well as metabotropic glutamate receptor type 5 (Cullen et al., 1997; Walsh et al., 2002; Wang et al., 2004; Klyubin et al., 2012). LTP deficits in APP/PS1 mice are also linked to disruption of the hippocampal pro-opiomelanocortin (POMC)/melanocortin 4 receptor (MC4R) circuit as the suppression of hippocampal MC4R activity exacerbated LTP impairments in these mice, and is alleviated by activation of the hippocampal MC4R-coupled Gs signaling and POMC/MC4R activity (Shen et al., 2016). MC4R activation also rescues amyloid-β-induced synaptic dysfunction thereby implicating the POMC/MC4R as a potential therapeutic target to rescue synaptic dysfunction in AD. Contextual fear conditioning deficits in aged 5XFAD mice is associated with different expression of hippocampal proteins than normal aging (Neuner et al., 2017). Neuronal depletion of calcium-dependent proteins in the dentate gyrus is tightly linked to AD-related cognitive deficits (Palop et al., 2003). Overall, these reflect amyloid-β’s multi-faceted disruption of processes involved in synaptic plasticity in AD.

Several studies have examined whether rescuing synaptic plasticity deficits in mouse models of AD can improve behavioral symptoms. Neuron-specific postnatal deficiency of PS1 prevented amyloid pathology and rescued LTP in AβPP_Ind_ mice but failed to prevent cognitive deficits observed in the object recognition test in these mice (Dewachter et al., 2002), suggesting that LTP deficits do not underlie all behavioral deficits in this model. Another approach of preventing LTP deficits in AD mice included activation of *Wnt* signaling as several studies have shown *Wnt* signaling activation to facilitate LTP in wildtype mice (Chen et al., 2006; Beaumont et al., 2007; Cerpa et al., 2011). Vargas et al. (2014) found that chronic activation of *Wnt* signaling enhanced basal excitatory synaptic transmission, facilitated LTP and improved episodic memory in APP/PS1 mice (Vargas et al., 2014). In attempts to rescue synaptic plasticity deficits in AD mice, Cissé et al. (2011) regulated NMDAR function using receptor tyrosine kinase EphB2 which phosphorylates NMDARs via Src-mediated tyrosine phosphorylation (Dalva et al., 2000; Henderson et al., 2001; Takasu et al., 2002; Chen et al., 2008; Cissé et al., 2011). The phosphorylation status of NMDAR subunits is correlated with cognitive performance, and levels of EphB2 and tyrosine-phosphorylated NMDARs are depleted in hAPP mice (Sze et al., 2001; Palop et al., 2005, 2007; Simón et al., 2009). Reversing EphB2 depletion in the dentate gyrus reversed LTP and memory impairments in hAPP mice (Cissé et al., 2011). Furthermore, neutralization of adenosine A_2__A_ receptors could restore associative CA3 LTP and revert memory deficits in APP/PS1 mice (Viana da Silva et al., 2016). Chronic intranasal administration of Colivelin (a novel and strong humanin derivative) reduced amyloid-β deposition in the hippocampus, rescued suppression of hippocampal LTP, and prevented AD-associated behavioral impairments in APP/PS1 mice (Wu et al., 2017). In addition, increasing levels of the secreted APPα (sAPPα, an alternative cleavage product of APP that has neuroprotective and neurotrophic properties) completely reversed deficits in LTP and spatial memory tasks in APP_S__we_/PS1ΔE9 mice (Tan et al., 2018). Therefore, it is important to keep in mind the protective roles of some biproducts of APP (for a review see Montagna et al., 2017), although the pathological biproducts of APP are often the focus in AD literature. Together these studies demonstrate potential for therapeutics that target LTP and its downstream pathways using a range of different methods to provide behavioral improvements. It is currently unknown whether these treatment strategies are applicable to idiopathic AD.

### Neurogenesis

Neurogenesis in the adult hippocampus is a dynamic process that continuously changes the dentate gyrus, and is important for hippocampal plasticity, learning, and memory (Altman and Das, 1965; Eriksson et al., 1998; Aimone et al., 2011; Gu et al., 2012). Adult hippocampal neurogenesis consists of three main stages: proliferation, differentiation, and survival (Dard et al., 2019). Controversy exists in the literature as to whether hippocampal neurogenesis is increased or decreased in mouse models of AD (Rodríguez et al., 2008; Demars et al., 2010; Hamilton et al., 2010; Krezymon et al., 2013; for reviews see Wirths, 2017; Dard et al., 2019). However, altered neurogenesis must still provide some cognitive benefit as conditional ablation of adult neurogenesis in APP_S__we_/PS1ΔE9 mice worsened behavioral performance in contextual conditioning and pattern separation tasks (Hollands et al., 2017). Together these studies suggest that deficits in adult neurogenesis may contribute to the pathology of AD, and points toward the possibility of increasing neurogenesis or using neural stem cells transplantation as an approach for preventing AD-associated changes in neuronal circuitry. In support of this idea Richetin et al. (2017) demonstrated that amplification of mitochondrial function rescued adult neurogenesis in APP/PS1 mice, and overexpression of the pro-neuronal marker Neurod1 increased dendritic growth and spine formation, and consequently rescued spatial memory in these mice (Richetin et al., 2017). Furthermore, neural stem cell engrafts into APP/PS1 mice were able to restore memory and promote endogenous neurogenesis and synaptic remodeling in these mice (Zhang et al., 2017).

### Changes in Neuronal Excitability, and Excitation/Inhibition Imbalance

AD-associated alterations have also been observed beyond the synapse, with hyperexcitability of hippocampal neurons observed both *in vitro* and *in vivo*. Hippocampal neurons show increased excitability in the 3xTg-AD model due to altered Kv2.1 potassium channel function (Frazzini et al., 2016). Similarly, neurons of aged SAMP8 mice are hyperexcitable and show altered voltage-dependent Ca^2+^ currents (Wang et al., 2017). In APP/PS1 mice hyperexcitability has been linked to structural degeneration of dendrites (Šišková et al., 2014). The dendritic structure is known to determine the electrical properties of neurons as it defines the input-to-output conversion, therefore, when dendritic integrity is impaired neuronal function is aberrant (Šišková et al., 2014). These *in vitro* demonstrations of hyperexcitability in AD were later confirmed *in vivo* in APP/PS1 mice using two-photon imaging in the hippocampus (Busche et al., 2012). Neuronal hyperactivity in the hippocampus *in vivo* was correlated with soluble amyloid-β levels (Busche et al., 2012). Together, these studies demonstrate that hippocampal hyperexcitability is a common feature of different mouse models of AD (for a review see Busche and Konnerth, 2015). In addition to alterations in neuronal excitability, mouse models of AD demonstrate deficits in γ-aminobutyric acid (GABA) pathways and altered excitation/inhibition balance leading to seizures. In APP/PS1 mice deficits in the GABAergic pathway and feed forward inhibition are age-dependent (Oyelami et al., 2016; Viana da Silva et al., 2019). In hAPP mice parvalbumin interneuron dysfunction and reduced levels of voltage-gated sodium channel subunit Nav1.1 have also been linked to abnormal oscillatory rhythms, network synchrony and cognitive function (Verret et al., 2012). Furthermore, APP/PS1 mice show somatostatin-positive interneuron axon loss, enhanced spine turnover, and impaired learning–dependent spine gain in association with memory deficits in these mice (Schmid et al., 2016). Similarly, soluble amyloid-β oligomers increase neuronal excitability by disrupting glutamatergic/GABAergic balance in the hippocampus, and this could be prevented by increasing GABA tone or partially blocking NMDAR activity (Lei et al., 2016). Moreover, APP/PS1 mice are also susceptible to seizures, the frequency of which is correlated with the load of amyloid-β plaques (Minkeviciene et al., 2009; Busche et al., 2012; Reyes-Marin and Nuñez, 2017). Seizure activity appears to trigger compensatory mechanisms in the dentate gyrus of hAPP mice as enhanced synaptic inhibition and GABAergic sprouting have been observed (Palop et al., 2007). Furthermore, synaptic and cognitive deficits in hAPP and APP23 mice are reversed by antiepileptic drugs which suppress neuronal network dysfunction (Bromley-Brits et al., 2011; Sanchez et al., 2012). Together these data show that deficits in inhibition leading to overexcitation and seizures is commonly seen in mouse models of AD and contributes to our understanding of epilepsy co-morbidity in AD patients.

Overall these studies show that inhibition is reduced in AD, which combined with hyperactive excitatory neurons massively shifts the ratio toward excess excitation leading to seizures (Figure 1), which negatively impact cognition. Increased excitability may eventually promote the excitotoxic damage observed in the AD brain. Therefore, restoration of excitation/inhibition balance may hold therapeutic potential in AD. The relation between the synaptic and plasticity changes to hyperexcitable networks seems counterintuitive as weaker synapses, impaired strengthening, and enhanced depression should lead to reduced network activity. However, changes in dendritic structure and activity levels can increase excitability of neurons leading to action potentials being triggered by fewer inputs. Other homeostatic changes, such as inhibitory alterations, that aim to restore activity levels may overcompensate and fail to restore balance.

### Astrocytic Changes

Alterations in glial function have also been observed in AD, and growing evidence shows that glial changes may precede neuronal changes and behavioral impairment noted in the progression of AD (Heneka et al., 2010; for a review see De Strooper and Karran, 2016). Astrogliosis is a universal feature of AD brains (Nagele et al., 2004; Rodríguez et al., 2009; Heneka et al., 2010, 2013). Inflammatory responses in glial cells contribute to the pathogenesis of AD, and several studies have highlighted specific therapeutic targets for the treatment of AD, such as targeting the inflammasome NLRP3 or RIPK1, an enzyme abundantly expressed in microglia (Heneka et al., 2013; Ofengeim et al., 2017). Pathological astroglial changes have been shown to be prevented by environmental enrichment in PDAPP-J20 transgenic mice (Beauquis et al., 2013). Additionally, it was identified that gamma frequency entrainment could recruit both glial and neuronal responses to attenuate AD-associated pathology (Iaccarino et al., 2016). Reactive astrocytes likely play a role in clearing damaged synapses and dendrites, however, they are limited in their ability to fully clear away debris (Gomez-Arboledas et al., 2018). The role of astrocytes in synaptic plasticity is also affected in AD (for a review see Singh and Abraham, 2017). Therefore, preventing glial pathology may represent a new therapeutic intervention for AD, and preventing abhorrent glial changes can be achieved by altering network activity, either naturally by changing the environment or artificially by stimulation.

### Changes in Brain Connectivity and Circuit Function

Brain connectivity and circuit function are disrupted in AD, in part due to synaptic and neuronal loss (Figure 1). At the synaptic level, amyloid-β induced LTD results in loss of dendritic spines (Hsieh et al., 2006; Wei et al., 2010). Amyloid-β-induced synapse loss and dendritic spine abnormalities have been noted by other studies in several mouse models of AD, such as the APP mice, APP/PS1 mice, PDAPP, and Tg2576 mice (Lanz et al., 2003; Spires et al., 2005; Shankar et al., 2007; Knafo et al., 2009). In hippocampal slice cultures from APP_SDL_ mice, spine loss was accompanied by changes in spine shape from mushroom to stubby spines (Tackenberg and Brandt, 2009; for a review see Tackenberg et al., 2009). Use of adeno-associated virus to express oligomeric amyloid-β in the hippocampus also resulted in spine loss (Forner et al., 2019). Interestingly, extracellular amyloid-β lead to a greater reduction in stubby spines than intracellular overexpression, while other spine types were equally affected (Forner et al., 2019). Amyloid-β pathology also results in dendritic abnormalities and atrophy. High-resolution confocal microscopy has revealed that, in the PSAPP mouse model of AD, dendrites passing within 40 μm of amyloid deposits displayed loss of dendritic spines, shaft atrophy, varicosity formation, and sprouting (Tsai et al., 2004; Grutzendler et al., 2007). Similarly, post-mortem human brains from AD patients also display similar dendritic alterations (Merino-Serrais et al., 2013), further emphasizing that amyloid deposits and their surroundings microenvironments are toxic to dendrites.

The hippocampal CA1 subregion is particularly more susceptible to AD-associated atrophy in comparison to CA2 or CA3 subregions (West et al., 2000; Frisoni et al., 2008; Apostolova et al., 2010). Selective neuronal death in brain regions most affected by AD has also been demonstrated in APP mice, and this was directly correlated with amyloid plaque formation (Calhoun et al., 1998). As a result, mouse models of AD demonstrate decreased functional connectivity within the hippocampus as well as the cortex, as examined by resting state fMRI and optical intrinsic signal imaging technique, respectively (Bero et al., 2012; Shah et al., 2013). Furthermore, functional coupling between the hippocampus CA1 region and medial frontal cortex is also impaired in mouse models of AD (Zhurakovskaya et al., 2019).

Spatial memory deficits in AD mice are attributed to changes in circuit function due to altered cellular responses in the hippocampus. At a cellular level, place cells play a critical role in spatial memory and these have been shown to be affected in AD. Place fields from control mice become spatially restricted and stable after repeated exposures of a new environment; however, APP mice produce a spatial code of lower resolution, reliability and accuracy (Zhao et al., 2014). Furthermore, hippocampal place cell degradation and MWM training deficits correlate with amyloid-β plaque burden, respectively in Tg2576 and PDAPP mouse models of AD (Chen et al., 2000; Cacucci et al., 2008). A lack of learning dependent changes in place cells in APP-PS1 mice has been correlated with impaired action-reward association tasks in a spatially defined environment (Cayzac et al., 2015). Impairments in rhythmic organization of place cell activity have also been observed in the 3xTg mouse model of AD, and may contribute to the unstable spatial representation and spatial memory deficits (Mably et al., 2017). Furthermore, in young rTg4510 mice high-frequency ripple oscillations and neuronal synchronization are reduced even though place fields of hippocampal CA1 cells are largely normal (Ciupek et al., 2015). Impaired cellular and network activity in the hippocampus therefore appear to contribute to spatial memory deficits in mouse models of AD.

Alterations in networks in other brain regions which are connected to the hippocampus are also observed in AD. For example cortical principle cells become hyperexcitable at the early stages of amyloid pathology, and via the thalamo-cortical pathway, drive thalamic cells too (Gurevicius et al., 2013; Busche et al., 2015). This precedes hippocampal electrophysiological abnormalities, and is hypothesized to underlie the network reorganization which leads to epileptic seizures (Palop et al., 2007; Minkeviciene et al., 2009).

### Treatment Strategies in Amyloid-β-Related AD

The prevention of behavioral deficits in AD mice has been studied extensively with a variety of different approaches: For example: Learning and age-related memory deficits can be prevented in APP/PS1, TgCRND8, Tg2576, and PDAPP mice with immunization against the amyloid-β peptide (Janus et al., 2000; Morgan et al., 2000; Dodart et al., 2002; Kotilinek et al., 2002). Such immunizations reduce pathological changes including plaque formation in PDAPP mice (Schenk et al., 1999). In 3xTg-AD mice, immunizations against amyloid-β have also been shown to act at the synaptic level by reducing synaptic impairments (Baglietto-Vargas et al., 2018). In this same AD mouse model, accumulation of intraneuronal amyloid-β precedes plaque and tangle pathology. Using immunotherapy to clear intraneuronal amyloid-β pathology rescued the early cognitive deficits seen in the MWM (Billings et al., 2005). Re-emergence of the amyloid-β pathology could again lead to cognitive deficits, implicating intraneuronal amyloid-β in the onset of cognitive dysfunction (Billings et al., 2005).

Despite billions of dollars being invested into drug development for AD, over 100 compounds have failed in clinical trials (Mehta et al., 2017). These potential disease-modifying drugs fall into four categories: monoclonal antibodies, gamma secretase inhibitors, tau aggregation inhibitors, and symptomatic treatments. Some examples of previously failed clinical trials include (i) bapineuzumab, one of the first monoclonal amyloid-β antibodies to reach phase 3 clinical trials, but unfortunately was found to have no significant clinical benefit (Salloway et al., 2014), (ii) solanezumab, which despite demonstrating an excellent safety profile and low incidence of vasogenic edema, failed to meet primary and secondary endpoints in the phase2B-3A study (Doody et al., 2014; Siemers et al., 2016), (iii) crenezumab did not show a significant benefit in treatment in comparison to placebo in a phase 2 trial (Miller, 2012), and (iv) gantenerumab did not meet a significant clinical efficacy endpoint in phase 3 trials at its administered dosage (Ostrowitzki et al., 2017). However, more recently, Aducanumab, a human monoclonal antibody that is selective for aggregated forms of amyloid-β has been examined as a potential treatment for amyloid-β-associated pathologies. *In vivo* multiphoton imaging of calcium homeostasis in aged Tg2576 mice demonstrated that acute topical application of aducanumab to the brain resulted in clearance of amyloid plaques, and chronic systemic administration ameliorated calcium overload and restoring intracellular calcium to control levels (Kastanenka et al., 2016). Aducanumab also restored NMDAR GluN1 and GluN2A subunit-expressing cell numbers to wildtype levels, thus indicating a potential restoration of neuronal network function and cognitive function in these mice. Phase I clinical trials using Aducanumab demonstrated an acceptable safety and tolerability profile of the drug, and it was shown to reduce amyloid deposition in the brain in a dose- and time-dependent manner (Ferrero et al., 2016; Sevigny et al., 2016). Phase III clinical trials were performed in 3200 individuals across 20 countries, but early analyses showed no promising effects of Aducanumab in decreasing amyloid burden or improving symptomology in patients and thus the study has been halted (Selkoe, 2019). However, longitudinal studies are required to investigate any potential long-term benefits of antibodies against amyloid-β.

Genetic, social, environmental, and pharmacological approaches have also been used to prevent behavioral deficits in AD models. For example, development of memory deficits was prevented in APP_S__we_/PS1ΔE9 mice by constitutive deletion of the amyloid-β-binding cellular prion protein (Gimbel et al., 2010). Conditional deletion of PrP^c^ at 12 or 16 months of age completely rescued MWM deficits, novel object recognition, and passive avoidance test in APP_S__we_/PS1ΔE9 mice, together with reversal of hippocampal synapse loss (Salazar et al., 2017). Memory deficits in APP/PS1 mice could be rescued by social interaction, and this effect was linked to increased levels of BDNF in the hippocampus (Hsiao et al., 2014). In addition, environmental enrichment led to reduced amyloid-β levels and amyloid deposition in APP_S__we_/PS1ΔE9 (Lazarov et al., 2005). Environmental enrichment also changes the function of microglia in a way that prevents their inflammatory response to human soluble amyloid-β oligomers (Xu et al., 2016). Recently it was demonstrated that environmental enrichment and voluntary exercise revives adult neurogenesis, reverses AD-associated memory deficits, and prevents amyloid-β seeding (representing early stages of plaque formation) via activated phagocytic microglia cells (Ziegler-Waldkirch et al., 2018). Therefore, prolonged environmental enrichment could protect against AD by regulating the brain’s innate immune system. 5xFAD mice displayed improved cognitive abilities, decreased amyloid plaque and neuroinflammation in the entorhinal cortex after treatment with RS67333, a partial 5-HT_4_R agonist, for 4 months (Baranger et al., 2017). Genetic reduction of tau expression has also been shown to prevent behavioral impairments and neuronal deficits (Roberson et al., 2007; Vossel et al., 2010). Similarly, expression of truncated versions of tau that lack dendritic localization has beneficial effects in APPswe transgenic mice (Ittner et al., 2010), fitting with evidence that shows the amyloid-β-induced mis-localization of endogeneous tau into dendrites is detrimental (Zempel et al., 2010). These are just a few of the treatment strategies that have shown promise in animal trials, however, there are not yet many that have translated well in human trials. However manipulating circuit function still holds promise for future treatments (for a review see Canter et al., 2016).

## Future Directions in Understanding Circuit Function in Amyloid-β-Related Alzheimer’s Disease

Given the complex multifaceted nature of the identified issues in AD it is becoming increasingly important to understand changes in brain networks *in vivo*. Examining circuit dynamics during behavior will give the next breakthroughs in our understanding. To date there have been several studies that have taken advantage of *in vivo* two-photon imaging to better understand circuit changes in the hippocampus and other brain regions. For example *in vivo* studies that have examined hyperactivity near plaques in both hippocampus and cortex (Busche et al., 2008, 2012) have identified significant heterogeneity in cell responses within the same brain region. In the visual cortex neuronal hyperactivity has been shown to affect function (Grienberger et al., 2012). Hyperactive neurons exhibited poor orientation tuning, which was correlated with impaired performance in visual-pattern discrimination (Grienberger et al., 2012). Furthermore, visual experience driven-expression of Arc is impaired in AD mice, providing further *in vivo* evidence of altered memory processes (Rudinskiy et al., 2012). Astrocytes in the cortex have also shown synchronous hyperactivity and intercellular calcium waves in APP/PS1 mice (Kuchibhotla et al., 2009). In future studies it will be crucial to understand the link between aberrant neuronal and glial activity *in vivo* in AD mice.

Examining *in vivo* dynamics of axons and dendrites longitudinally with disease progression and/or treatment also offer promise for understanding complex changes in AD models. For example, long-term imaging revealed how axon pathology proceeds around amyloid-β plaques in APP-PS1 mice (Blazquez-Llorca et al., 2017). Axons near plaques appeared swollen before becoming disconnected, over a time course of several months (Blazquez-Llorca et al., 2017). In addition, instability of dendritic spines and axonal boutons near plaques was revealed in this way and could be prevented by γ-secretase inhibitor treatment (Liebscher et al., 2014). Further studies are needed to reveal not just structural but also functional changes in dendrites and axons using calcium and voltage imaging *in vivo*. Furthermore *in vivo* imaging in freely moving animals using miniaturized microscopes is another exciting possibility for future studies (for a review see Werner et al., 2019).

Activation of specific subsets of neurons using channel rhodopsin is also an important approach to understand circuit changes in AD. By utilizing learning-dependent expression of channel rhodopsin it is possible to label memory engram cells (Ryan et al., 2015). It is then possible to re-activate these cells ontogenetically and trigger memory retrieval (Ryan et al., 2015). This approach has been used to restore fear memory in young AD mice (Roy et al., 2016). More studies are required to increase our understanding of the specific pathways involved in memory deficits in AD so that treatments can be targeted to the right networks at the right time in disease progression.

In conclusion, hippocampal deficits in synapse and neuronal function manifest into behavioral abnormalities in mouse models of AD. However, more research and consensus in the field are required to completely understand hippocampal deficits in AD. With the increasing development of *in vivo* recording techniques in awake and freely moving animals, future studies will extend our current knowledge about how hippocampal function is altered in AD by combining network imaging with behavior. It will be crucial to identify network changes early and treat them before pathology becomes widespread. However, because network changes likely contribute to disease progression this could lead to future treatments that prevent AD symptoms from worsening.

## Author Contributions

YV, JM, and JC wrote the manuscript.

## Conflict of Interest

The authors declare that the research was conducted in the absence of any commercial or financial relationships that could be construed as a potential conflict of interest.
